# Forest Densification and Pollution Rival Climate in Driving US Tree Demographic Change

**DOI:** 10.1111/gcb.71124

**Published:** 2026-10-01

**Authors:** Matthew M. Kling, P. Alexander Burnham, Matthew P. Dube, Brian J. McGill, Bailey P. McLaughlin, Stephanie N. Miller, Timothy M. Waring, Nicholas J. Gotelli

**Affiliations:** ^1^ Department of Biology and Gund Institute for Environment University of Vermont Burlington Vermont USA; ^2^ Department of Biology University of Vermont Burlington Vermont USA; ^3^ Department of Computer Information Systems University of Maine at Augusta Augusta Maine USA; ^4^ School of Biology and Ecology, Mitchell Center for Sustainability Solutions University of Maine Orono Maine USA; ^5^ School of Economics, Mitchell Center for Sustainability Solutions University of Maine Orono Maine USA

**Keywords:** climate change, demography, density dependence, forest ecology, growth, mortality, pollution, recruitment

## Abstract

Forests play critical roles in biodiversity, climate regulation, and human well‐being, yet their responses to simultaneous environmental changes remain poorly understood. Across the United States, forest demographic rates are influenced by climate change, atmospheric pollution trends, and systematic changes in forest density. In spite of long‐term interest in these facets of global change, their relative importance has not previously been quantified and compared. Here, we apply a framework that decomposes demographic trends into sensitivity (species' responsiveness to environmental variables) and exposure (magnitude of environmental change experienced). Using nationwide forest inventory data from 2002 to 2024, we analyzed how recruitment, growth, and mortality of 100 tree species responded to six environmental variables representing climate, pollution, and forest density. This analysis showed that, despite substantial research attention on climate impacts, changes in forest density and atmospheric pollutant deposition (particularly sulfur) showed stronger associations with all three demographic rates, driven by both higher exposure and greater sensitivity to these factors. We also found that, of the demographic processes, recruitment probability was the most sensitive to each of the six environmental drivers, emphasizing the importance of regeneration in shaping forest responses to global change. The results also document a general slowdown in forest dynamics over recent decades, with declining recruitment, growth, and mortality on average across the United States, albeit with substantial geographic and taxonomic variation. This comprehensive analysis provides a framework for understanding forest responses to complex environmental change, identifying the most influential drivers of demographic processes, and developing targeted conservation and management strategies in an era of rapid change.

## Graphical abstract

Forests across the contiguous United States are responding to simultaneous changes in climate, atmospheric pollution, and forest density, yet the relative importance of these drivers for tree demographic rates has rarely been compared. Using two decades of nationwide forest inventory data, we quantified the sensitivity of recruitment, growth, and survival in 100 tree species to six environmental drivers, and combined these sensitivities with observed rates of environmental change to predict demographic trends. Despite intense research focus on climate change, we found that declining sulfur deposition and increasing forest density had stronger associations with demographic change than climate, driven by both greater species sensitivity and larger exposure to these factors. Recruitment probability was the most environmentally sensitive demographic rate across all six drivers, highlighting regeneration as the primary demographic filter through which environmental trends affect forests. Together, these results suggest that pollution recovery and forest densification have been the dominant shapers of recent US forest demographic change.

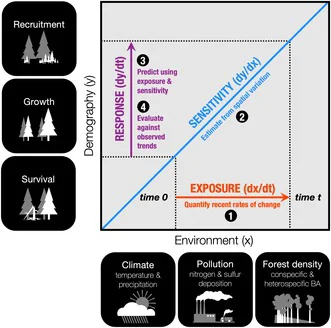

## 1 Introduction

Global environmental trends are driving widespread ecological change, including in forests, which play critical roles in biodiversity, climate, and human well‐being (Trumbore et al. 2015; Millar and Stephenson 2015; McDowell et al. 2020). Changes in climate, pollution, land use, biotic interactions, and disturbance regimes influence forests through their effects on three fundamental demographic processes: recruitment of new trees, growth of existing trees, and mortality of existing trees. Collectively, the processes of recruitment, growth, and mortality determine the dynamics of density, biomass, species composition, and range shifts of forests over recent and coming decades (Clark et al. 2016; Franklin et al. 2018; Zhu et al. 2014). Understanding how humans are impacting these ecological dynamics is critical for biodiversity conservation and natural resource management but has proven challenging for several reasons.

A first challenge is that although there is substantial research on various drivers of change, studies typically focus on single categories of driver in isolation, with a particularly large body of work focused on climate impacts (Anderegg et al. 2015, 2020). Climate change affects forest demography through multiple pathways. Warming and altered precipitation regimes influence recruitment through changes in seedling establishment conditions, affect growth through extended or contracted growing seasons, and increase mortality via drought stress (Allen et al. 2015; Anderegg et al. 2015). Climate change also contributes to shifting patterns of disturbance, including fire, pests, and other disturbance agents (Westerling 2016; Cohen et al. 2016), that alter forest demography and can lead to broader forest reorganization (Seidl and Turner 2022). But climate is not the only human‐induced driver of changing forest dynamics. Atmospheric pollution, particularly nitrogen and sulfur deposition, alters soil chemistry and nutrient cycling, stimulating growth in nitrogen‐limited systems, but potentially reducing recruitment and increasing mortality in areas experiencing soil acidification or nutrient imbalances (Bobbink et al. 2010; Thomas et al. 2010). In addition, widespread increases in forest density represent another major anthropogenic trend shaping North American forests. Driven by post‐agricultural reforestation, fire suppression, and recovery from historical timber harvesting, these density increases alter demography through intensified resource competition, typically reducing recruitment and individual tree growth while potentially accelerating mortality through competitive exclusion (Oliver and Larson 1996; Bradford and Bell 2017). These various drivers rarely act in isolation, as forests are generally exposed to multiple stressors whose effects can be additive, multiplicative, or antagonistic (Bytnerowicz et al. 2007; Trumbore et al. 2015). Despite these known impacts, the relative importance of climate, atmospheric pollution, and forest density trends remains poorly quantified and, with limited exceptions (e.g., Dietze and Moorcroft 2011; Canham and Murphy 2017), has not been systematically compared among species.

Second, although demographic processes together comprise the “demographic niche” that represents where populations can succeed and determines forest responses to environmental change (Holt 2009; Evans et al. 2016; Pang et al. 2026), they are typically studied in isolation. Recruitment determines potential range shifts and community composition (Zhu et al. 2014), growth drives carbon storage and forest structure (Franklin et al. 2018), and mortality influences forest turnover and resilience (McMahon et al. 2019), among many other demographic effects. Previous work has extensively documented each process individually, but rarely examines all three together, which limits our ability to predict overall forest responses (Clark et al. 2021).

Addressing these challenges requires a framework that can systematically compare multiple drivers, examine multiple demographic processes in parallel, and bridge spatial and temporal scales. The “exposure‐sensitivity” framework (Dawson et al. 2011; Williams et al. 2008; Clark et al. 2021) addresses these needs by decomposing environmental responses into two components: exposure (the magnitude of environmental change) and sensitivity (the per unit change in a demographic response variable for a given environmental change, which could represent a positive or negative outcome). The total response, conceptually calculated as their product, represents each driver's overall effect. This decomposition enables quantitative comparisons across drivers, species, geographies, and demographic processes, and is particularly valuable when strong responses may stem from either high sensitivity or large changes in driver values.

Here, we apply this exposure‐sensitivity framework to analyze forest demographic changes across the contiguous United States. We use forest plot data from the US Forest Inventory and Analysis (FIA) spanning two decades (2002–2024) to examine how recruitment, growth, and mortality of 100 tree species respond to six environmental variables representing climate change, air pollution, and forest density (Figure 1). Using a GLM implementation of the exposure‐sensitivity framework, we first estimate species' demographic sensitivities by combining spatial variation in environmental conditions with demographic rates quantified from repeat plot measurements; while these sensitivity estimates are correlational, spatial gradients can be viewed as natural experiments that reveal demographic responses to environmental variation. We then combine these sensitivities with temporal environmental changes (exposures) over this same recent time period to predict demographic responses. The focus on contemporary changes reduces uncertainty in future exposure estimates, and enables evaluation of predictions against observed demographic time series (Dietze et al. 2018; Norberg et al. 2019).

**FIGURE 1:**
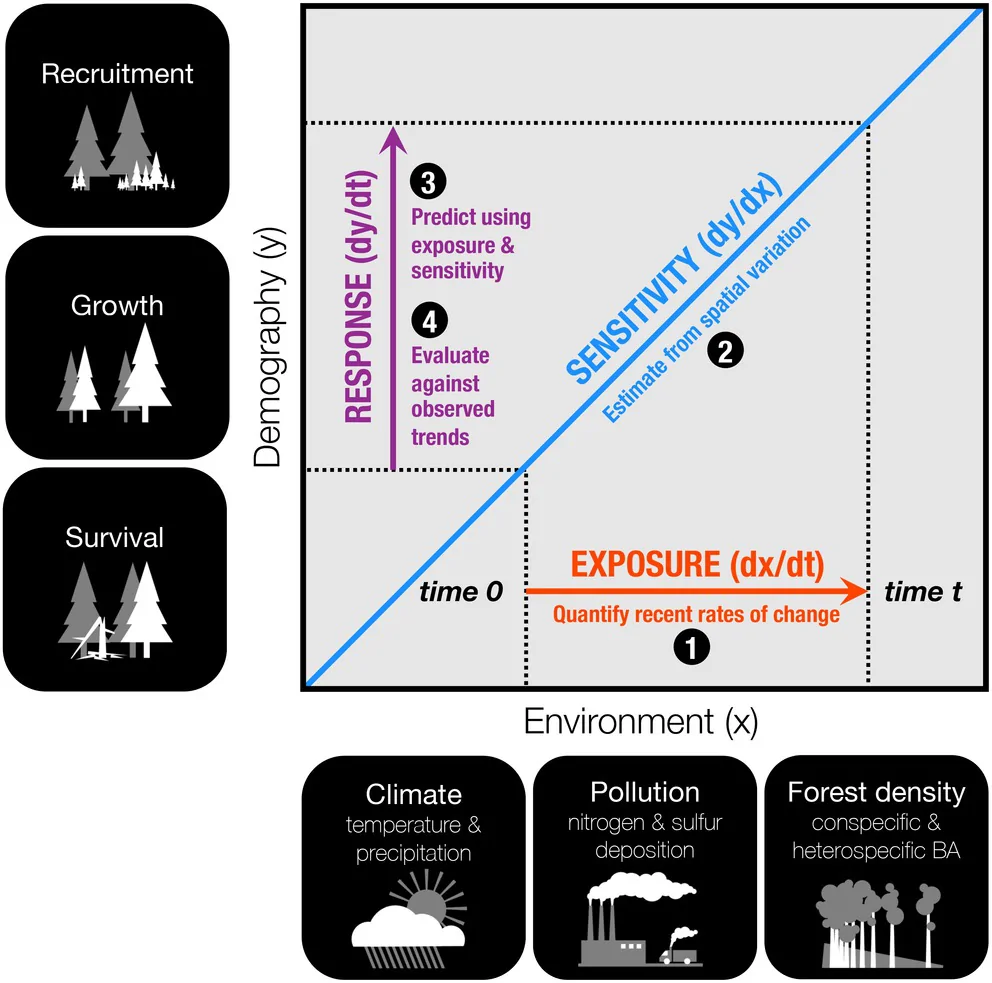
Conceptual and analytical framework linking environmental change to forest demography. The *x*‐axis represents environmental conditions (e.g., climate, pollution, forest density), and the *y*‐axis represents demographic rates (recruitment, growth, and survival). (1) Exposure (*dx*/*dt*) quantifies recent temporal change in each environmental variable. (2) Sensitivity (*dy*/*dx*) is estimated from spatial variation in demographic rates across environmental gradients. (3) The demographic response (*dy*/*dt* ≈ *dy*/*dx* × *dx*/*dt*), calculated by combining exposure and sensitivity across all environmental variables, predicts how vital rates are expected to have changed over time. (4) Predicted responses are then evaluated against observed demographic trends from forest inventory data over recent decades.

Our analysis addresses two key questions: First, across US forests, how do different components of the tree life cycle respond to environmental change? And second, what drives the variation in these responses—is it primarily variation in sensitivity to particular environmental drivers, variation in the rates of change in those drivers, or both? The answers have implications for understanding future forest trends, setting monitoring priorities, and developing management strategies in an era of rapid environmental change.

## 2 Materials and Methods

### 2.1 FIA Data

Our analyses are based on data from the US Forest Service Forest Inventory and Analysis program for the contiguous United States (Bechtold and Patterson 2005), accessed in June 2025. The FIA is a standardized survey of hundreds of thousands of randomly located forest plots across the United States, re‐surveyed periodically on multiyear intervals. Individual trees are tracked across surveys, enabling monitoring of recruitment, growth, and mortality rates over time. Our analysis is based on plots surveyed from 2002 through 2024, each surveyed between two and five times, with more remeasurements in the eastern United States (Figure S1). Data from all plots were used in all analyses, with the exception of one model validation analysis described below that required plots surveyed at least three times.

We combined data from the “plot,” “subplot,” “condition,” and “tree” tables, excluding plots that were inventoried only once and plots with any recorded planting or harvesting activities. Our analyses focus on the 100 most common tree species in the dataset, which collectively represent 91% of forest basal area across the contiguous United States; the remaining rarer species were excluded due to insufficient data for reliable model fitting.

Our analysis is focused on trees with a diameter at breast height (DBH) > 1 in., as smaller trees are not fully tracked in the FIA program. Adult trees with DBH over 5 in. are inventoried on subplots, saplings with DBH of 1–5 in. are inventoried on smaller microplots nested within each subplot, and exceptionally large trees are additionally inventoried on larger macroplots in some geographic regions. Our analyses include all three size categories.

### 2.2 Demographic Rates (Response Variables)

For each FIA remeasurement interval (the period spanning two consecutive inventories) for a given plot, we calculated growth and mortality rates for every individual tree, and recruitment rates for every species with adults present.

Growth was calculated as the annual growth rate (AGR), defined as AGR = (*d*_1_/*d*_0_)^1/*t*^ − 1, where *d*_0_ and *d*_1_ are the measured DBH values from the initial and subsequent inventories, respectively, and *t* is the number of years between inventories. Growth rates were square root transformed prior to statistical analysis to make relationships with DBH approximately linear.

Mortality over a survey interval was recorded if a tree was explicitly reported as changing from live to dead across the two surveys, or if it implicitly died due to being recorded in the pre‐interval survey but not the post‐interval survey.

Recruitment was defined as the appearance of a new sapling (DBH 1–5 in.) during the survey interval. While many FIA studies model recruitment as counts per unit land area (e.g., Zhu et al. 2015), we estimated recruitment per unit conspecific adult basal area (CBA) (e.g., Qiu et al. 2021) to align with classical matrix demography and with our other per‐adult vital rates. We expressed the rate as recruits per m^2^ CBA per year by normalizing recruits and CBA per hectare and dividing by census interval. Because recruitment rate per unit conspecific basal area is undefined for plots lacking conspecific adults—which is the case for roughly a third of recruits—we used targeted smoothing of adult basal area values to enable inclusion of these ecologically important recruitment events. Discarding these recruits would bias results toward common species and closed‐canopy conditions, while arbitrarily setting CBA to a small nonzero value would introduce artifacts. Instead, we resolved this using a k‐nearest‐neighbor smoothing approach in environmental space: for each species, zero‐adult plots with recruitment were matched to their nearest nonzero‐adult plot in standardized environmental space (using the six predictors described below), and both plots' CBA values were replaced by their mean. This approach assumes that individual plots are noisy samples of recruitment processes determined by local environmental conditions, such that averaging across environmentally similar plots with and without conspecific adults provides a principled estimate of expected CBA. This is essentially the same assumption that underlies our regression models themselves (described below), which ensures internal consistency between the smoothing procedure and the demographic analyses.

### 2.3 Environmental Data (Predictors)

For every plot, we obtained data on long‐term means and year‐over‐year trends in climate, pollution, and forest density variables.

Climate data were extracted from annual temperature and precipitation rasters from CHELSA v2.1 (Karger et al. 2017, 2021) for the years 2000–2019 at the location of every FIA plot. While FIA adds noise to the true locations of plots for privacy reasons, the scale of this “fuzzing” roughly matches the 1 km grid resolution of the climate data, so these values are expected to be reasonable estimates of plot macroclimate (Gibson et al. 2013). Prior to analysis, we log‐transformed precipitation values to improve normality and ecological relevance.

To evaluate whether our conclusions were sensitive to the choice of climate variables, we repeated the full analysis using an alternative pair of variables intended to reflect drought and heat extremes rather than mean conditions. These were the annual minimum of monthly climatic moisture index (CMI, calculated as precipitation minus potential evapotranspiration) and the annual maximum of monthly vapor pressure deficit (VPD), both derived from CHELSA monthly time series for the same period. These two variables replaced mean annual temperature and precipitation, with all other aspects of the analysis held constant, so that the two parameterizations are directly comparable.

Pollution rates representing annual total bias‐adjusted sulfur and nitrogen deposition for the years 2002–2019 were extracted at each plot's location from 12 km resolution Community Multiscale Air Quality (CMAQ v5.3.2) rasters produced by the US EPA's EQUATES project (US Environmental Protection Agency 2021; Benish et al. 2022). Pollution rates were log‐transformed prior to analysis to make spatial patterns less skewed and temporal trends more linear.

Forest density was calculated using the FIA data described above. We calculated CBA and heterospecific basal area (HBA) separately for each combination of species, subplot, tree, and survey year. For a given focal tree, HBA is the total cross‐sectional stem area of trees of other species, while CBA is the stem area of other trees of the same species (not including the focal tree). To account for differences in the tree size classes inventoried across different plot types, we calculated CBA and HBA separately at the microplot, subplot, and macroplot levels, converted these to normalized densities in units of m^2^ ha^−1^, and then summed these densities across the levels (modeling demographic rates as a function of current forest density follows standard practice in population ecology, where per‐capita rates are expressed as functions of conspecific and heterospecific abundance, as in logistic and Lotka‐Volterra models; our sensitivity estimates derive from variation in vital rates across forests spanning a wide range of densities and successional states).

For the growth and mortality models, DBH was used as a predictor in addition to the six environmental variables described above. For a given tree, DBH was calculated as the average of pre‐ and post‐interval diameters. DBH values were log‐transformed to reduce skew and make observed relationships with growth and mortality more linear.

Prior to model fitting, all environmental variables were standardized across the dataset. This standardization was performed separately for each species to reflect the scale of spatial environmental variation across their individual geographic ranges.

For each plot, we retained environmental data from the year 2000 through the year of the most recent plot inventory (or through the most recent year in which environmental data was available). These time series data were used to calculate long‐term environmental averages for model fitting, and temporal environmental trends for model prediction.

### 2.4 Model Fitting (Sensitivity)

For each of the three demographic processes, we fit a Bayesian regression model for each species, predicting demographic rates as a function of spatial environmental variation in long‐term mean environment.

Growth was modeled using the ordinary multiple linear regression model:

$$y\sim N\left(\mathrm{x\beta},\sigma \right)$$

$$\beta_{0}\sim N\left(0,10\right);\beta_{j}\sim N\left(0,1\right)\;\text{for}\;j\geq 1$$

$$\sigma \sim N^{+}\left(0,0.1\right)$$

where y is a vector of square root transformed annual growth rates, x is a matrix of predictor data including the seven variables described above as well as an intercept term, and β is a vector of eight inferred parameters estimating the effects of each predictor on growth, and σ is the residual standard deviation.

Mortality was modeled using a logistic GLM. To infer annual mortality rates from inventory data spanning multiyear survey intervals, we used the model form:

$$m=1/\left(1+e^{-\mathrm{x\beta}}\right)$$

$$p=1-{\left(1-m\right)}^{t}$$

$$y\sim \text{Bernoulli}\left(p\right)$$

$$\beta_{0}\sim N\left(0,10\right);\beta_{j}\sim N\left(0,1\right)\;\text{for}\;j\geq 1$$

where *m* is a vector of annual mortality probabilities, *p* is the probability of mortality over a survey interval of *t* years, y is a binary vector indicating whether a tree died over a given survey interval, x is a matrix of predictor data as described above, and β is a vector of inferred parameters representing environmental effects on mortality.

Recruitment was fit as a hurdle model, with a logistic GLM representing the probability of nonzero recruitment and a lognormal GLM representing recruitment rate conditional on there being nonzero recruitment, with each component influenced separately by the predictors. Hurdle and zero‐inflated mixture models are commonly used in tree recruitment studies (Fortin and DeBlois 2007; Zhang et al. 2012), due to theoretical hypotheses that different processes influence the probability and quantity of recruitment, as well as statistical necessities arising from the bimodal, zero‐inflated recruitment distributions that many species (including most in our data set) exhibit. While many studies use Poisson or negative binomial distributions in hurdle models, we used a lognormal distribution because our recruitment variable is continuous. This model can be written as:

$$\theta_{a}=1/\left(1+e^{-x\beta_{\theta }}\right)$$

$$\theta =1-{\left(1-\theta_{a}\right)}^{t}$$

$$p\left(y=0\right)=1-\theta$$

$$y\;|\;y>0\sim \text{Lognormal}\left({\mathrm{x\beta}}_{\mu },\sigma_{\mu }\right)$$

$$\beta_{0}\sim N\left(0,10\right);\beta_{j}\sim N\left(0,1\right)\;\text{for}\;j\geq 1$$

$$\sigma_{\mu }\sim N^{+}\left(0,1\right)$$

where $\theta_{a}$ is the annual probability of nonzero recruitment, θ is the corresponding probability over a survey interval of *t* years, y is the observed recruitment rate, x is a matrix of predictor data as described above, $\beta_{\theta }$ is a vector of inferred coefficients representing environmental effects on $\theta_{a}$, $\beta_{\mu }$ is a vector of inferred coefficients representing environmental effects on y when y>0, and $\sigma_{\mu }$ is the residual standard deviation of log recruitment rate when y>0.

Spatial autocorrelation is undoubtedly present in these data, and would need to be accounted for with hierarchical modeling if inference (statistical significance testing of individual parameters) were our focus. However, nonhierarchical models are sufficient in our case since our focus is on prediction and on broad macroecological patterns, goals that do not depend on inferring parameter uncertainty for individual species (Legendre 1993).

Regression was done in a Bayesian framework using Stan (Carpenter et al. 2017). Each model (*n* = 300) was run using five separate MCMC chains, each with 1000 warmup iterations and 1000 sampling iterations. Sampler behavior was evaluated using effective sample size to assess mixing, the r‐hat statistic to assess convergence, and divergent transitions to assess model degeneracy.

### 2.5 Environmental Trends (Exposure)

For each plot, species, and environmental predictor variable, we fit a linear model regressing the predictor value against year to quantify rates of environmental change.

For temperature and precipitation, we used linear regression based on annual time series from 2000 to 2019 (the final year of availability for the climate dataset we used). For nitrogen and sulfur deposition, we used annual time series data from 2002 to 2019; because deposition trends were estimated on the log scale, we used a two‐group slope estimator (the difference in mean log deposition between the second and first halves of the record, divided by the difference in their mean years).

For forest density variables, we used linear regression based on data from FIA inventory years. The slopes of these models represent exposure, that is, the rate of observed environmental change experienced by trees on a given plot. To compare relative exposure magnitudes across variables with different units, slope units were standardized relative to spatial variation across the entire FIA dataset.

### 2.6 Predicted Demographic Trends (Response)

Leveraging space‐for‐time substitution (STS), we used the fitted demographic models in combination with exposure data to predict rates of change in each of the three demographic variables in each FIA plot.

First, for every plot we used the linear environmental exposure models described above to calculate predicted values of every environmental variable for two “evaluation” years. We used the years 2009 and 2010, but since the environmental trend models are log‐linear or linear, other years would yield similar or identical predicted rates of change in demographic rates. These predicted environmental values capture rates of change in the mean environment over the ~20‐year study period rather than annual variation found in data from individual years; this is important since tree demography responds to time‐integrated environmental conditions, and since FIA data also integrate over multiple years due to the periodic inventory schedule.

Next, we used these environmental values, in combination with the parameters from the demography models for each species, to calculate estimated demographic rates for each plot and tree in the dataset for each of the two evaluation years.

Finally, rates of modeled demographic change were calculated as the difference between these two time points. To capture uncertainty in parameter estimates, trends were calculated using multiple draws from the posterior distribution and then averaged.

### 2.7 Variable Importance

We summarized these sensitivity, exposure, and response measures for each tree across the dataset to assess the relative importance of each environmental variable to modeled demographic trends. We used two separate approaches: a relative magnitude approach and a variance partitioning approach.

For the relative magnitude approach, we compared the relative magnitudes of the six environmental drivers in influencing a given trend component (sensitivity, exposure, response). To enable comparison across environmental variables and demographic rates with disparate units, we standardized all effect sizes across the data set based on the standard deviations of the dependent and independent variables. For each environmental variable for each tree, we took the absolute value of the trend component; we then calculated the mean of these absolute values across all trees in the dataset to determine the mean absolute standardized effect (MASE). Finally, we converted these into relative magnitudes by scaling them by the maximum magnitude observed across the variables and demographic rates for a given trend component. Note that the three trend components each vary across different margins of the dataset. For a given environmental variable and demographic process, sensitivity values are constant across plots within a species, while exposure values are constant across species within a plot. Response values vary within species and plots.

For the variance partitioning approach, we asked, for a given demographic rate, how much of the variation in total modeled response was driven by exposure and sensitivity of each of the environmental variables. We used a hierarchical variance partitioning method implemented in the R package “rdacca.hp” (Lai et al. 2022) to quantify how much of the variation in the modeled response across all trees in the dataset was attributable to each of 12 predictors (exposure and sensitivity for each of the six environmental variables). This approach quantifies how correlated the modeled demographic responses are with each predictor while accounting for correlations among the predictors, allocating both unique and shared variation to individual predictors by averaging each predictor's marginal contribution across all possible combinations of variables.

To assess whether the relative importance of environmental drivers was consistent across the major east–west biogeographic divide of US forests, we repeated both variable importance analyses separately for plots east and west of 100° W. This longitudinal boundary falls within a region of sparse forest cover and few FIA plots.

### 2.8 Model Evaluation

We evaluated models for goodness of fit to the training data, and for the predictive accuracy of temporal demographic trends calculated using space‐for‐time substitution (STS). Goodness of fit was measured using the coefficient of determination (*R* ^2^) between observed and predicted means across prediction deciles, after grouping trees into decile bins based on their predicted values and calculating the average observed and predicted demographic rate within each bin. This decile‐based approach provides a consistent measure across all models, including the logistic components where traditional *R* ^2^ is not applicable, and evaluates model performance at an ecologically relevant scale by assessing mean predicted demographic rates across groups of trees rather than stochastic demographic events in individual trees (Figure S2).

To evaluate STS predictive performance, we compared observed to predicted rates of change in demographic rates. While predicted rates of change can be calculated for each individual tree, changes in observed demographic rates can only be calculated at an aggregate level by pooling data from many trees. This analysis used the subset of FIA plots surveyed at least three times—the minimum necessary to observe a change in rates—most of which are in the eastern United States (Figure S1). We began by calculating the observed rate of change in each demographic variable for each species and subplot. We used the same linear regression approach described above for environmental trends, fitting a linear model to measured demographic rates and using it to predict fitted values for 2009 and 2010. The difference between these values gives the observed rate of change in a demographic variable for each species and plot.

To assess predictive accuracy for a given demographic rate, we compared these observed versus predicted rates across various levels of the dataset (Figure S3). For each species, we calculated the within‐species correlation between observed and predicted rates of change, as well as the mean observed and predicted rates of demographic change. We also calculated the correlation among these species‐level means and fit linear regressions of predicted versus observed species‐level means. All these summaries were weighted by the number of tree‐years represented by each record.

In addition to the species level, we also calculated these summary statistics for geographic blocks, with block defined as all FIA plots in a given grid cell with a resolution of three degrees longitude and latitude (Figure S1). Lastly, we calculated these same summaries after binning observations into deciles of the predicted rate of demographic change.

All analysis were done in R (R Core Team 2025) and Stan (Carpenter et al. 2017). Analysis code and results are available at https://doi.org/10.5281/zenodo.22774454 (Kling et al. 2026).

## 3 Results

### 3.1 Exposure to Environmental Change

Over the past two decades, forests across the United States experienced substantial changes in multiple environmental drivers (Figure 2). To compare the magnitudes of exposure across variables with differing units, we calculated observed rates of change after normalizing each variable relative to its spatial variation across all FIA plots in the data set; we report absolute values of these rates. Among the six variables we examined, sulfur deposition exhibited the highest exposure, with a standardized rate of 1.41 standard deviations per decade—more than three times the next fastest‐changing variable, nitrogen deposition (0.40 sd/decade). Changes in forest density (conspecific and heterospecific basal area) and precipitation occurred at intermediate rates (0.28, 0.28, and 0.23 sd/decade, respectively), while temperature exhibited the slowest rate of change (0.074 sd/decade) relative to its spatial variation.

**FIGURE 2:**
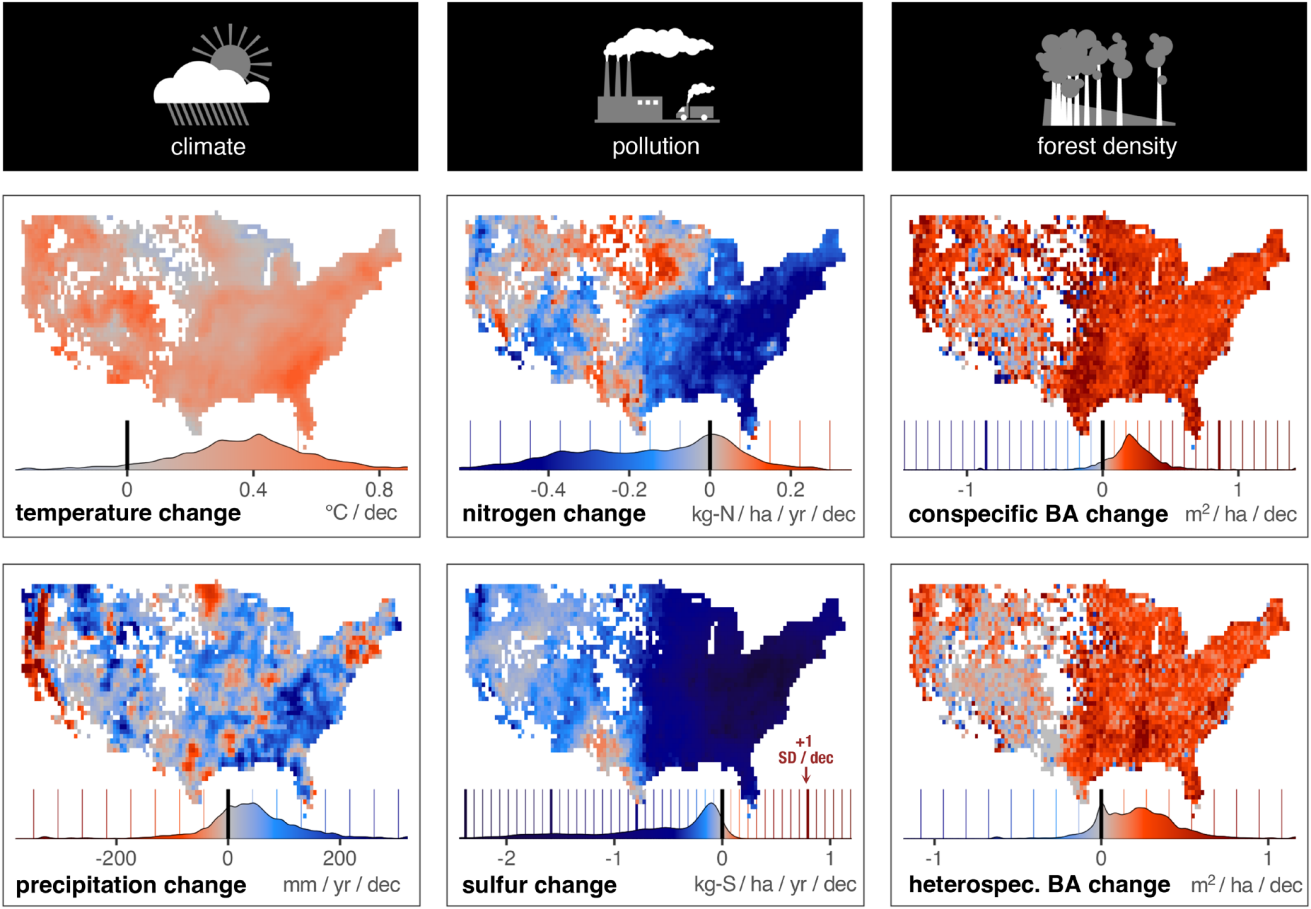
Exposure of forest trees to recent environmental trends. Maps and histograms show the slopes of regression lines fit through environmental time series from ~2000 to 2020, in units of change per decade. The histogram axes give rates of change in native units for each variable, while color scale and histogram gridlines represent rates of change in standardized units shared across all variables (calculated by dividing rates of change by the standard deviation in each variable's long‐term mean across all trees in the data set). The values in each grid cell are averages of the slopes for individual trees in the FIA plots within the cell.

There was substantial spatial heterogeneity in both the direction and magnitude of exposure across variables. Temperature trends showed widespread warming, while precipitation trends were more variable across regions. Sulfur and nitrogen deposition declined substantially across the eastern United States over the study period but had more variable trends in the West. Forest density increased in most of the eastern United States and Pacific Northwest, with particularly rapid increases in the Southeast and along the Pacific coast, while trends in the interior West were more spatially heterogeneous (Figure 2).

### 3.2 Sensitivity of Demographic Rates

Our sensitivity results comprise a set of standardized regression coefficients from a multiple linear model fit for each species and demographic rate, based on spatial variation across plots. We found that environmental drivers affect demographic processes differently. In some cases, effects were relatively consistent across species, while in other cases there was high variability in effect size and direction across species (Figure 3). Of the demographic rates, recruitment probabilities were most sensitive to overall environmental variation as assessed with mean absolute standardized effects (MASE), with the largest MASE for all six environmental variables (Figure 5a).

**FIGURE 3:**
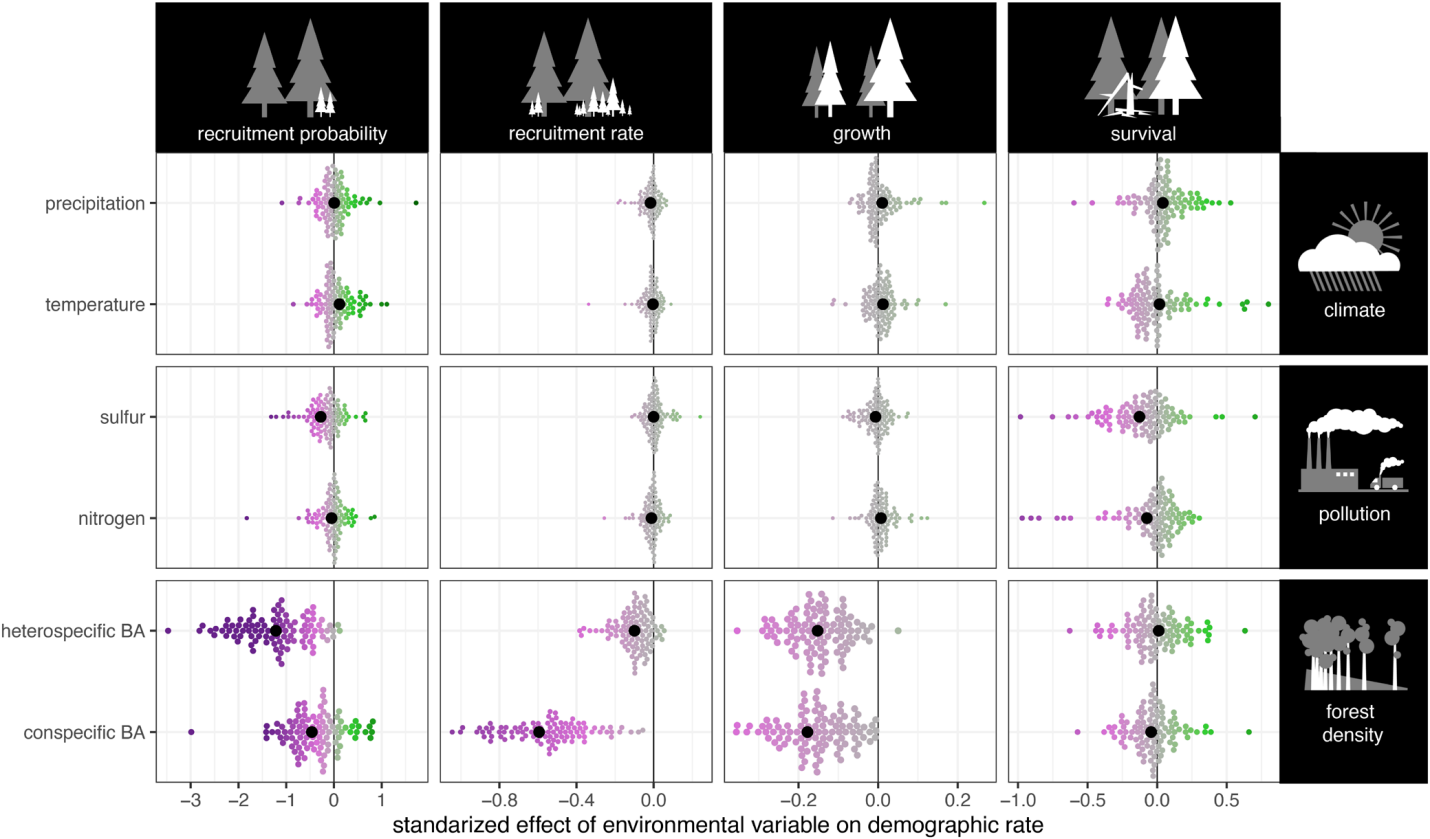
Sensitivity of demographic rates to spatial environmental variation. Plots show distributions of effect sizes across tree species, with colored points representing the posterior mode for each individual species (colored by the effect) and black points representing the overall abundance‐weighted mean effect. Effect sizes are standardized for dependent and independent variables across the full data set.

We modeled recruitment sensitivity as the separate effects of environmental variables on the probability of recruitment and on the rate of recruitment when it occurred. Overall, recruitment was most sensitive to forest density (Figures 3 and 5a), with both conspecific basal area (CBA) and heterospecific basal area (HBA) negatively associated with overall recruitment in most species. However, the mechanisms differed markedly between these two predictors despite being negative in both cases, with CBA acting primarily through recruitment rate and HBA primarily through recruitment probability. Sulfur deposition was also negatively associated with recruitment on average, primarily via its relationship with recruitment probability, though the effect was variable across species. Nitrogen deposition and climate variables had inconsistent, albeit sometimes strong, associations with recruitment. Across species, standardized effect sizes were generally stronger for recruitment probability than for recruitment rate, except for CBA, which had similarly strong negative relationships with recruitment rate (Figures 3 and 5a).

Growth rates were also more strongly associated with forest density than with other environmental predictors. Among species, higher CBA and HBA were almost universally associated with slower growth rates, with CBA having slightly stronger effects than HBA in the average species. Growth sensitivity to climate and pollution variables showed greater interspecific variation, but on average, growth was faster in warmer parts of a species' range and areas with lower sulfur deposition (Figures 3 and 5a).

In contrast to growth and recruitment, survival was most sensitive to pollution and climate variables. Annual survival rates tended to be higher in plots with lower sulfur deposition and greater precipitation. Temperature and nitrogen deposition also showed strong associations with survival, but the direction of these effects was highly variable among species (Figures 3 and 5a).

### 3.3 Modeled Demographic Responses

Based on the combination of sensitivity estimates and observed environmental trends, models predicted increasing survival but declining recruitment and growth over recent decades for the majority of species and landscapes (Figure 4). Specifically, modeled recruitment rates declined for the median FIA plot in 65% of landscapes (0.5 degree grid cells) and 62% of species, modeled growth declined in 67% of grid cells and 63% of species, and modeled survival increased in 66% of grid cells and 70% of species.

**FIGURE 4:**
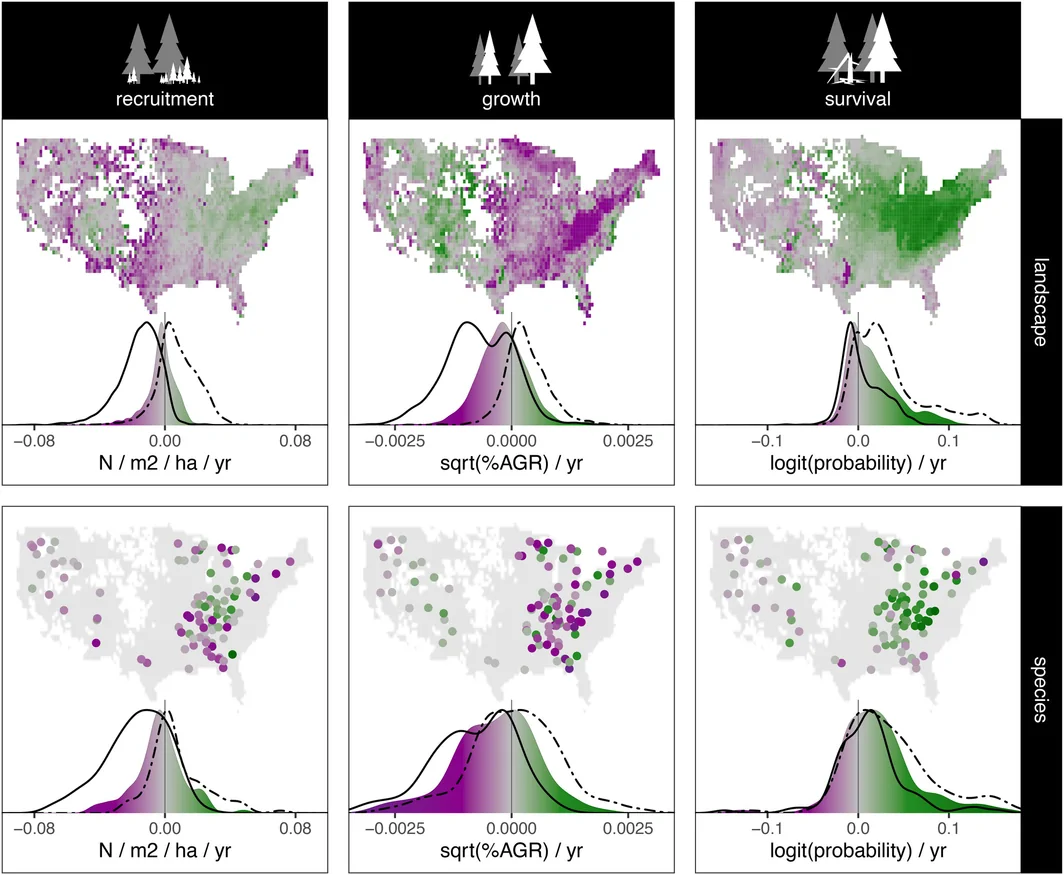
Modeled trends in forest demographic rates across focal species in the FIA dataset from ~2000 to 2020. In the maps and histograms, color indicates the median predicted rate of change across all FIA plots in an individual landscape (grid cell) or across all plots occupied by a given species (illustrated with points placed at the geographic centroid of the plots it occupies). Black curves on the histogram plots show distributions of the 25th (solid) and 75th (dashed) percentiles within each landscape or species. The tails of some curves are not shown, to better visualize the central patterns.

Modeled trends in all three demographic rates exhibited strong geographic patterns (Figure 4). Projected trends across the eastern United States were characterized by decreasing growth and increasing survival. In contrast, trends were more heterogeneous for these demographic rates across the western United States, and for recruitment across the full study area. There was substantial heterogeneity in projected trends within landscapes and grid cells. Nearly every grid cell and species encompassed both increases and decreases in each of the three demographic rates.

We found that variation in both exposure and sensitivity contribute strongly to variation in demographic trends—depending on the environmental variable and demographic process, one, the other, or both change components may predominate (Figure 5). While the strongest modeled responses were characterized by both high exposure and high sensitivity (e.g., sulfur deposition effects on survival rates and recruitment probabilities), there were other cases when strong responses arose primarily from sensitivity (e.g., heterospecific forest density relationship with recruitment rate) or exposure (e.g., sulfur effects on recruitment rate).

**FIGURE 5:**
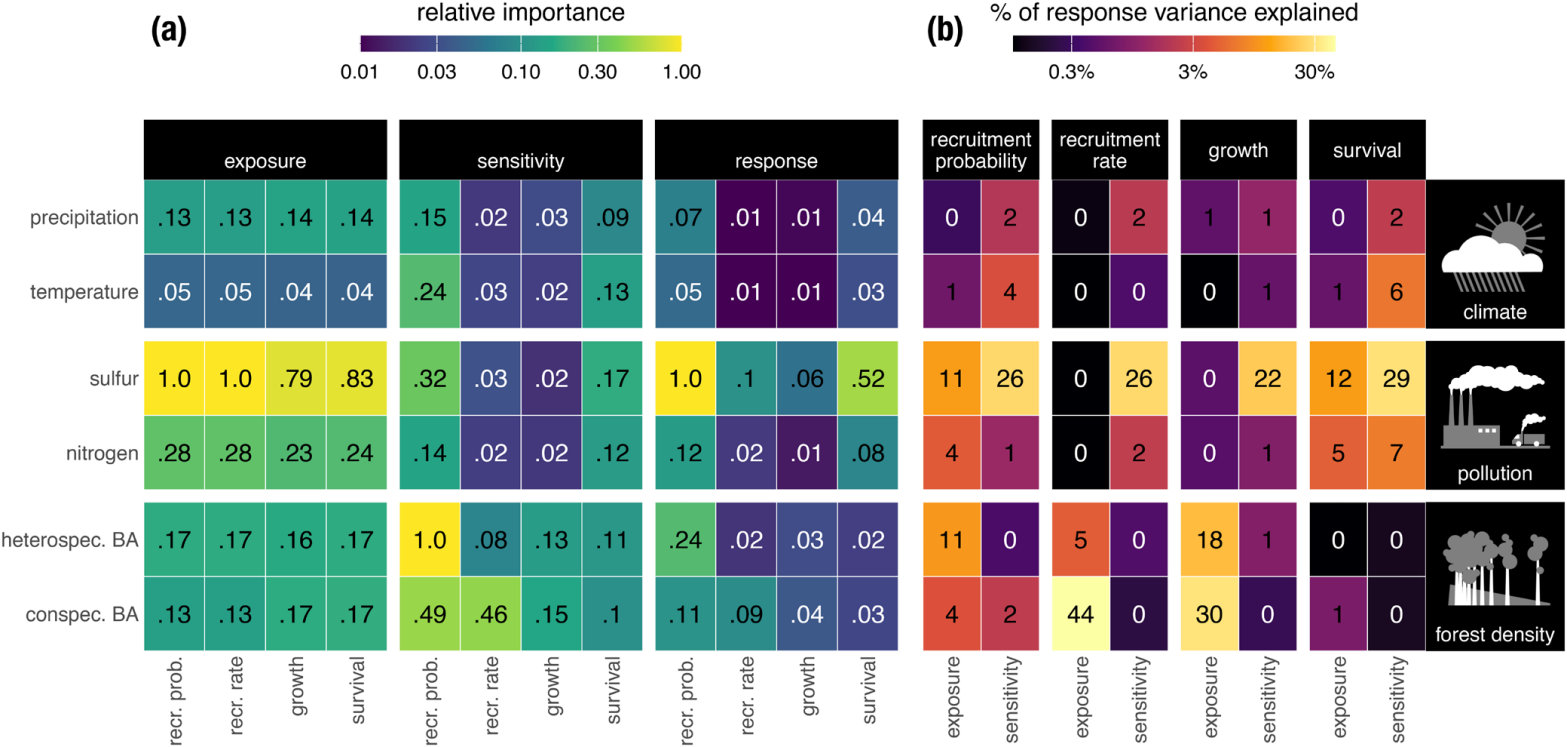
Heatmaps showing the relative importance of environmental variables in driving modeled recent demographic trends. (a) Variable importance based on mean absolute effect sizes; these are fully standardized and are scaled relative to the maximum for each change component (exposure, sensitivity, response), so can be compared within each component. (b) Proportion of modeled response variation explained by variation in each driver and component; the 12 values depicted under each of the four demographic rates, plus unexplained variance (illustrated in Figure S4), sum to 100%. Colors are on a log scale.

For a given species and plot, the product of sensitivity and exposure to a given environmental variable gives that variable's contribution to the modeled trend in a demographic rate, with the absolute value of this response representing the strength of the contribution. Modeled trends in all four demographic rates were driven most strongly by sulfur pollution (Figure 5a). Recruitment probabilities had stronger modeled responses to every one of the six environmental variables than did any of the other three demographic rates (Figure 5a).

Variance partitioning analyses (Figure 5b) showed that overall variation in modeled demographic trends was correlated primarily with variation in exposure to forest density and variation in sensitivity to pollutant deposition. Specifically, the components that explained more than 10% of the variation in modeled demographic trends included exposure to conspecific density changes (explaining 30% of variation in growth trends and 44% for recruitment rate trends), exposure to heterospecific density changes (explaining 18% of growth trend variation) and sensitivity to sulfur deposition (explaining 22%–29% of variation in each of the four demographic rates). Exposure and sensitivity to climate variables were not strongly correlated with modeled trends in any of the demographic processes; the strongest climate association was with sensitivity of mortality to temperature, which explained ~6% of variation in modeled mortality trends.

These results were qualitatively unchanged under an alternative parameterization using climate variables representing drought and heat extremes (Figure S5). Climate was a somewhat more important driver in this parameterization: standardized exposure to annual maximum VPD change was roughly twice that of mean annual temperature, and recruitment probability was more sensitive to both extreme climate variables than to their mean climate counterparts. Nonetheless, climate remained the weakest of the three driver categories in both the relative magnitude and variance partitioning analyses, with sulfur deposition and forest density continuing to dominate modeled demographic trends.

Geographically stratified analyses showed that the prominence of sulfur deposition was greatest in the eastern United States but that sulfur was also an important factor in the West (Figure S6). The east–west asymmetry was driven mainly by exposure rather than sensitivity—standardized sulfur exposure was roughly an order of magnitude lower in western than in eastern plots, whereas sulfur sensitivity was lower by roughly half. Across western plots alone, variation in sulfur sensitivity remained the leading correlate of variation in modeled survival trends, accounting for 22% of total variance (compared with 28% in the East). Modeled trends in western growth and recruitment rate were instead dominated by exposure to conspecific basal area change (36% and 77% of variance, respectively).

### 3.4 Model Evaluation

This analysis offers the opportunity to assess the practice of space‐for‐time substitution (STS) that has been widely used in global change ecology to compensate for the deficit of longer observational time series. Our results offer modest support for STS, with demographic models based on spatial environmental variation capturing the average direction of observed temporal trends as well as variation around these averages, but leaving a substantial portion of variation unexplained (Figure S3). For all three demographic processes, the predicted mean direction of change matched observed trends. For tree survival, this predicted mean trend was very close to the observed trend, while for growth and recruitment, model predictions substantially underestimated observed rates of change.

STS was also modestly successful at predicting variation in demographic trends both within and among species (Figure S3, first row). Across the three demographic processes, between 73% and 99% of species showed positive within‐species correlations between predicted and observed trends across plots. Correlations among species‐level mean predicted and observed trends were also significantly positive for all three processes (*r* = 0.23–0.68). At the spatial block level, 78%–99% of blocks exhibited positive within‐block correlations, with mean‐trend correlations of *r* = 0.42–0.78 across processes (Figure S3, second row).

## 4 Discussion

Unlike most forest demography studies that examine narrow sets of drivers and individual demographic processes, our nationwide analysis simultaneously evaluated the influences of climate, pollution, and forest density on tree recruitment, growth, and mortality. Using an exposure‐sensitivity framework applied to two decades of forest inventory data, we found that US forests are exposed to a complex mosaic of environmental changes and that tree demographic rates exhibit distinct sensitivities to these changes. Our findings reveal several key patterns, with implications for understanding and managing forests in an era of rapid environmental change.

In contrast to the emphasis on temperature and precipitation in many global change studies (e.g., Allen et al. 2015; McDowell et al. 2020; Clark et al. 2016; Zhu et al. 2014), we found that recent systemic changes in pollution and forest basal area were more strongly associated with tree growth, survival, and recruitment than was climate change. This was true both in our primary analysis focused on mean temperature and precipitation, as well as in an alternative version focused on trends in climate extremes (Figure S5). While these findings are consistent with prior work in the forest ecology literature demonstrating the importance of pollution and competition (Horn et al. 2018; Fenn et al. 2020; Thomas et al. 2010; Caspersen et al. 2000), few prior studies have systematically compared temporal trends in these variables as global change drivers, with some important exceptions focused exclusively on sensitivity and on individual demographic rates (Canham and Murphy 2016a, 2016b, 2017; Dietze and Moorcroft 2011). Our results suggest that pollutant deposition and forest density trends should be considered more prominently in ecological forecasts and management strategies.

However, our finding that climate change was a comparatively weak driver of recent demographic trends does not mean that it is unimportant for forest change more broadly. Although warming had the smallest average effect on individual demographic rates in our analysis, its ubiquity across nearly all sites was a striking contrast with spatially variable trends in other variables. This pervasive exposure suggests that systemic changes in forest composition, such as the thermophilization of US tree communities (Rosenblad et al. 2023), may arise not from particularly strong sensitivity or exposure, but instead from the widespread nature of climate exposure patterns. Even modest demographic responses, when acting consistently across species and landscapes, can drive large‐scale directional shifts over time. In addition, our analysis may not fully capture demographic responses to extreme climate events that are increasingly recognized as important drivers of forest mortality and recruitment failure (Allen et al. 2015; Anderegg et al. 2015). While our secondary climate parameterization captured long‐term trends in the intensity of VPD and CMI extremes and the frequency of years experiencing them, it still did not directly model the demographic consequences of individual extreme events, which would require a different modeling framework and empirical dataset.

It is also likely that the observed effects of recent temperature trends have been partially buffered by increases in forest density. Studies in Europe have demonstrated that secondary forest regrowth can cool the understory microclimate, offsetting macroclimate warming trends and potentially mitigating warming effects on juvenile trees (De Frenne et al. 2019; Zellweger et al. 2020). The widespread increases in forest density that we document across the eastern United States are likely having a similar effect, though this buffering capacity may diminish in the future as density nears carrying capacity.

Looking beyond recent climate trends to potential future forest changes, it is likely that the importance of climate will increase relative to other drivers. Climate change is predicted to continue unabated for decades even under optimistic emissions scenarios, in contrast with recent pollution and forest density trends, which often represent ongoing recovery from legacy changes and are likely slowing considerably in many places while continuing to progress in others. Climate could also increase in importance if nonlinearities emerge as climate departs further from historic regimes. Still, the fact that climate is both the environmental driver for which we have the most data on projected future change, and also the one with the smallest modeled demographic impact in our study, highlights a need for better future scenarios data for pollution and land cover change.

Among the non‐climatic drivers we analyzed, atmospheric pollution showed particularly strong associations with tree recruitment and growth. The strong relationship with sulfur deposition in our analysis reinforces and extends longstanding evidence that acid precipitation has shaped US forests in profound and lasting ways. Many prior studies have documented negative effects of sulfur deposition on tree growth and survival (Thomas et al. 2010; Horn et al. 2018; Fenn et al. 2020), but few have examined these effects across demographic processes or at continental scales. Our results suggest that even after decades of declining emissions, sulfur remains one of the most important correlates of demographic change in forests across the United States. This likely represents both non‐negligible ongoing deposition rates as well as ecological persistence of historical pollutant loads (Lawrence et al. 2015). While the sulfur deposition story is largely driven by eastern forests where historical deposition rates (and sample sizes) were highest, our geographically stratified variable importance analysis clarifies that sulfur is also an important factor in western forests, particularly for survival and recruitment probability. In contrast to sulfur, nitrogen deposition showed more heterogeneous associations with demographic rates, consistent with its dual role as both a nutrient and a stressor (Bobbink et al. 2010; Canham and Murphy 2017; Horn et al. 2018; Van Houtven et al. 2019). Together, these findings suggest that despite widespread assumptions that pollution effects are mainly historical, pollution legacies remain a leading factor in US forest dynamics and should remain a central consideration in forest research and management.

Our results on forest basal area effects suggest that historical land use legacies continue to shape contemporary forest dynamics through density‐dependent competition. The widespread increases in forest density we document—driven by agricultural abandonment, recovery from past harvesting, and fire suppression—align with classic competition theory and with empirical work demonstrating negative density‐dependent effects on tree performance (Oliver and Larson 1996; Canham and Murphy 2016a, 2017; Zhu et al. 2015). We expand this prior work by separating the distinct effects of conspecific and heterospecific density on multiple demographic processes and recruitment components. Our finding that growth rates declined with increasing basal area of both conspecifics and heterospecifics, with slightly stronger effects for conspecifics, aligns with theoretical and empirical expectations of greater intraspecific resource overlap (Horn et al. 2018; LaManna et al. 2016). For recruitment, our hurdle model highlights contrasting mechanisms, revealing that recruitment *probability* (which emphasizes fecundity and seedling establishment) is more sensitive to heterospecific density, while recruitment *rate* (which more strongly reflects seedling growth and survival) is more sensitive to conspecific density. This pattern aligns with classic Janzen‐Connell effects in which conspecific adults reduce juvenile success via host‐specific pathogens and herbivory (Comita et al. 2014). This same pattern also reflects the dual, countervailing role of conspecifics in enhancing seed rain while reducing seedling success (e.g., Peters 2003; Ramage et al. 2017). In future work, approaches that more explicitly separate fecundity from seedling establishment and survival (e.g., Clark et al. 2021; Sharma et al. 2022) could help to further clarify the contrasting roles of conspecific and heterospecific density. Together, these findings suggest that the ongoing increases in forest density we document across large parts of the United States could systematically dampen growth and regeneration potential by shifting the demographic bottlenecks faced by trees.

These density and pollution effects show marked geographic variation. Most forests across the eastern United States saw increasing density and decreasing pollution over recent decades, while trends in western forests were more variable. Combined with sensitivity values, the result for overall responses is a clear trend toward decreasing growth and increasing survival in the East, consistent with recovery from historical pollution and land use (Duveneck et al. 2017). In contrast, western forests showed more heterogeneous or even opposite demographic trends, consistent with the region's more variable drought and disturbance regimes. This spatial divergence aligns with previous findings that western forests are more closely tracking climate change, while eastern forests are constrained by greater demographic inertia associated with legacy effects and slower turnover (Clark et al. 2021; Duveneck et al. 2017; Sharma et al. 2022). Our results extend this east–west asymmetry to incorporate the effects of multiple environmental drivers on processes representing the complete demographic life cycle.

Looking across all these driver effects, our inclusion of the complete life cycle highlights two notable findings. First, for all six environmental variables, recruitment probability had the highest sensitivity (measured in standardized effect sizes) of the four demographic rates we examined. This suggests that the seed production and establishment processes that underlie recruitment probabilities represent the primary demographic filter where environmental trends show their strongest associations. Although the environmental sensitivity of recruitment has been documented (Dobrowski et al. 2015; Zhu et al. 2015; Clark et al. 2021), recruitment has received less study than growth and mortality, and comparisons of the three processes remain rare. Our systematic analysis across six environmental variables and 100 species provides a comprehensive quantification of recruitment sensitivity relative to other demographic rates, confirming its role as the most environmentally responsive component of the tree's life cycle.

A second key finding that emerged from assessing the full life cycle is that on average, US forests saw declines in recruitment, growth, and mortality, implying a general deceleration of demographic turnover driven by all three demographic rates in concert. While previous studies have reported individual components of this trend—such as reduced growth and recruitment in aging, late‐successional stands (Oliver and Larson 1996), or lower mortality rates following reductions in nitrogen and sulfur deposition (Fenn et al. 2020)—our continent‐wide synthesis is among the first to document all three demographic processes shifting in ways that collectively imply a slowdown in forest turnover.

This observed demographic slowdown is driven primarily by forests across the eastern United States (which represent the majority of FIA plots), and likely reflects the combination of two mechanisms mentioned above. First, stand maturation and increasing forest density may reduce demographic rates by intensifying competition and canopy closure, limiting regeneration and growth while stabilizing survivorship in late‐successional cohorts (Franklin et al. 2018; Das et al. 2016). Second, improvements in air quality—particularly declining sulfur deposition—may be contributing to increased survival across many species and regions. These interacting legacies of land use and environmental policy appear to have dampened forest dynamics over recent decades. Although climate change is amplifying disturbance regimes and mortality risk in some landscapes (Allen et al. 2015), our results suggest that the most immediate demographic consequences of recent environmental change have been decelerative, rather than destabilizing.

While this finding is tangential to our primary goal in this study, which was to explore the environmental drivers of demographic trends rather than to report observed demographic changes themselves, it merits discussion here, as well as further investigation, due to its potential consequences for the adaptive capacity of tree communities. High rates of recruitment and mortality promote evolutionary and ecological responses to environmental change by increasing turnover, enabling selection to act, and facilitating compositional shifts through colonization and replacement. In contrast, reduced demographic flux, for example, due to stand maturation, suppressed recruitment, or increased survival, can constrain these adaptive processes by effectively “locking in” existing forest structure and composition. In this light, legacy effects of air pollution recovery and land‐use history may represent a double‐edged sword: while they have improved tree survival and stabilized forests in the short term, they may slow the turnover required for future migration or local adaptation (Aitken et al. 2008).

Several limitations should be considered when interpreting our results. First, our analysis is correlational, and our sensitivity estimates may partly reflect unmeasured factors that covary with our predictors across space; the mechanistic interpretations we offer should be read as hypotheses warranting further testing rather than as demonstrated causal effects. Second, because FIA does not inventory trees in sparsely populated woodlands and our study did not include relatively rare tree species, our findings primarily represent common forest tree species. Third, while our analysis was comprehensive in many respects, it did not incorporate potentially important anthropogenic factors such as CO_2_ fertilization effects (Keenan et al. 2013; Walker et al. 2021), disturbance regimes like fire (Stevens‐Rumann et al. 2018), or biotic agents such as pathogens (Lovett et al. 2016). And finally, our models did not account for potential nonlinear effects of environmental drivers or complex interactions and feedbacks among them; although our parallel analysis using drought and heat extreme metrics (Figure S5) indicates that our conclusions are not an artifact of representing climate by annual means, the demographic consequences of individual extreme events are not fully captured by our analysis. The substantial unexplained variance in observed temporal trends suggests these and other factors not included in our models may play important roles. Future research could address these limitations by incorporating additional drivers and exploring more complex relationships among them. Studies that explicitly model nonlinear responses and interactions between climate, pollution, and forest density would be particularly valuable. Additionally, although we focused on exposure and sensitivity components of vulnerability, adaptive capacity represents an important third component that operates on longer timescales than those addressed in our study (Beever et al. 2016). Future work that incorporates measures of adaptive capacity, such as genetic diversity or phenotypic plasticity, would complement our findings and provide a more complete picture of forest vulnerability to environmental change. And while FIA is a singularly valuable data source for studying forest dynamics, longer‐term monitoring and more frequent remeasurement of forest plots would also improve our understanding of forest responses to environmental change.

Our evaluation of space‐for‐time substitution (STS) methods also yielded important insights. While STS has been the subject of debate in the ecological literature and previous studies have reported mixed conclusions regarding its performance (Evans et al. 2025; Blois et al. 2013; Lovell et al. 2023), our results provide a relatively rare large‐scale test of the approach. For all three demographic processes, we found positive correlations between predicted and observed demographic trends across species, geographic locations, and predicted trend deciles, though these explained only a modest fraction of observed variation (Figure S3). Interestingly, we also found that observed demographic changes generally outpaced model predictions. This is surprising, since one might expect ecological inertia to cause observed changes to lag behind predictions. One potential explanation is that populations are locally adapted to their baseline environments, a pattern that is common in trees (Savolainen et al. 2007). This could cause populations to have steeper response functions than the species as a whole, and thus respond more rapidly than predicted by STS. Alternatively, the faster‐than‐predicted observed responses could also reflect synergistic interactions among simultaneously changing variables, or nonlinear responses to environmental variables as they move beyond historical ranges. These findings suggest that while space‐for‐time substitution can provide directionally accurate predictions of forest responses to environmental change, it may underestimate the magnitude of actual changes, highlighting both the value of the approach and the importance of calibrating predictions against observed temporal trends. While STS has enough predictive power to be useful in the absence of other analyses, more directly measuring temporal response is desirable when possible.

In summary, this analysis provides several important insights for understanding and managing forests under global change. First, changes in atmospheric pollution and forest density are more strongly associated with recent tree demographic change than is climate change, suggesting that forest responses to environmental change cannot be fully understood through a climate‐centric lens. Second, recruitment probability is the most environmentally sensitive demographic process, making regeneration dynamics essential for predicting forest compositional shifts. And third, the demographic slowdown we document may constrain forests' adaptive capacity by reducing the turnover necessary for evolutionary and ecological responses to future change. Together, these findings underscore the importance of monitoring multiple environmental drivers, prioritizing recruitment dynamics in forecasting efforts, and recognizing that legacy effects may be as consequential as ongoing climate trends in shaping future forests.

## Author Contributions

**Matthew M. Kling:** conceptualization, methodology, formal analysis, writing – original draft, visualization. **P. Alexander Burnham:** writing – review and editing. **Matthew P. Dube:** writing – review and editing, funding acquisition. **Brian J. McGill:** conceptualization, writing – review and editing, project administration, supervision, funding acquisition. **Bailey P. McLaughlin:** conceptualization, writing – review and editing. **Stephanie N. Miller:** conceptualization, writing – review and editing. **Timothy M. Waring:** writing – review and editing, funding acquisition, supervision. **Nicholas J. Gotelli:** conceptualization, methodology, writing – review and editing, project administration, supervision, funding acquisition.

## Funding

This work was supported by National Science Foundation (2019470).

## Conflicts of Interest

The authors declare no conflicts of interest.

## Supporting information


**Figure S1:** Map of FIA plots used in the study, colored by the number of times each plot was surveyed. All plots shown here were used for most analyses. For the analysis evaluating model performance in space‐for‐time substitution, only plots that had been inventoried three or more times (primarily located in the eastern United States) were used; in one analysis these were grouped into spatial blocks consisting of three‐degree grid cells, illustrated here in black.
**Figure S2:** Model fit to training data for each of the three demographic rates. In the scatterplots, every species is represented by a red point indicating its overall mean, and by a black line connecting the 10 predictive deciles. The histograms show distributions of species‐level correlations between observed and predicted rates across individual trees (*r*, which includes uncertainty form individual‐level demographic stochasticity) and coefficients of determination across predictive deciles (*R* ^2^, which averages over individual stochasticity to represent population‐level demographic rates); red lines indicate *r* and *R* ^2^ across species means.
**Figure S3:** Results of model evaluation for space‐for‐time substitution, in which observed recent demographic trends (*x*‐axis) were compared to trends predicted by demographic models parameterized on spatial patterns in long‐term environmental means (*y*‐axis). Each point represents the mean observed and predicted change for a species (first row), a three‐degree spatial block (second row; see Figure S1 for map), or a decile of the distribution of predicted probabilities (third row), with point size indicating the relative amount of data in each group, and color indicating the correlation between predicted and observed trends across trees within the group. Green lines and shaded regions show linear fits and their 95% confidence intervals across points, while dashed lines are one‐to‐one references. Annotations are as follows: CS, percentage of points with the correct sign (i.e., falling in the first or third quadrants); *p*, *p* value of this correlation; PC, percentage of groups with positive correlations across trees within the group; *r*, correlation across points. Axes for recruitment are shown on a pseudo‐log scale (linear near zero, logarithmic beyond) to accommodate the wide range of rates; the green linear fits therefore appear curved.
**Figure S4:** Results of variance partitioning analysis explaining dataset‐wide variation in modeled demographic responses (rates of change) as a linear function of variation in sensitivity and exposure. Each of the four rows represents a separate analysis, partitioning the total variation in predicted trends in the demographic rate listed at right into 13 categories (sensitivity and exposure for each of the six environmental variables, as well as unexplained variation).
**Figure S5:** Relative importance of environmental variables in driving modeled demographic trends, from a parallel analysis using an alternative set of climate variables reflecting drought and heat extremes (annual minimum monthly climatic moisture index [CMI] and annual maximum monthly vapor pressure deficit [VPD]) instead of mean annual temperature and precipitation. All other aspects of the analysis are identical to those underlying Figure 5. (a) Variable importance based on mean absolute effect sizes. (b) Proportion of modeled response variation explained by each variable and component, from variance partitioning analyses; the 12 values depicted under each demographic rate, plus unexplained variance, sum to 100%. Colors are on a log scale. Exposure to maximum VPD is roughly twice that of mean annual temperature, and recruitment probability is more sensitive to these extreme metrics than to mean climate; nonetheless, climate remains the weakest of the three driver categories overall. Note that the two extremeclimate variables are more strongly correlated with each other (*r* = −0.68) than are temperature and precipitation (*r* = 0.08), so their individual contributions in (b) are more diluted by covariation; climate is thus best compared between parameterizations as a category.
**Figure S6:** Relative importance of environmental variables in driving modeled demographic trends, computed separately for western and eastern plots. The top row of panels shows results for western plots (west of 100° W) and the bottom row for eastern plots (east of 100° W). (a) Variable importance based on mean absolute effect sizes; values are scaled relative to a single maximum per change component (exposure, sensitivity, response) across both regions, so they are comparable across regions as well as within components. (b) Proportion of modeled response variation explained by variation in each driver and component, from variance partitioning analyses conducted separately within each region; the 12 values depicted under each demographic rate, plus unexplained variance, sum to 100%. Total variance explained differs among regions and demographic rates (ranging from 41% for western survival to 83% for western growth), so values in (b) are most directly compared within rather than across panels; the standardized effect sizes in (a) do not have this limitation. Because the FIA dataset is dominated by eastern plots, results for the East are similar to the combined results presented in Figure 5. Colors are on a log scale.

## Data Availability

The code, model outputs, and derived data supporting this study are available in Zenodo at https://doi.org/10.5281/zenodo.22774454 (Kling et al. 2026). This study used the following publicly available secondary data. Forest inventory data were obtained from the USDA Forest Service Forest Inventory and Analysis Database version 1.5.1.07, accessed in June 2025. Climate data were obtained from CHELSA v2.1 (Karger et al. 2021; https://doi.org/10.16904/envidat.228). Atmospheric deposition data were obtained from the US EPA's bias‐adjusted annual total deposition estimates (2002–2019) derived from the EQUATES CMAQv5.3.2 simulations (US EPA 2021; https://doi.org/10.15139/S3/F2KJSK), accessed in August 2023.
