# Supplementary material for: Forest Densification and Pollution Rival Climate in Driving US Tree Demographic Change
Source: Glob Chang Biol. 2026 Oct 1;32(10):e71124. doi: 10.1111/gcb.71124 (PMC13628142; doi:10.1111/gcb.71124)
Supplement: Supplementary file 1 [file GCB-32-e71124-s001.pdf]

SUPPLEMENTARY INFORMATION

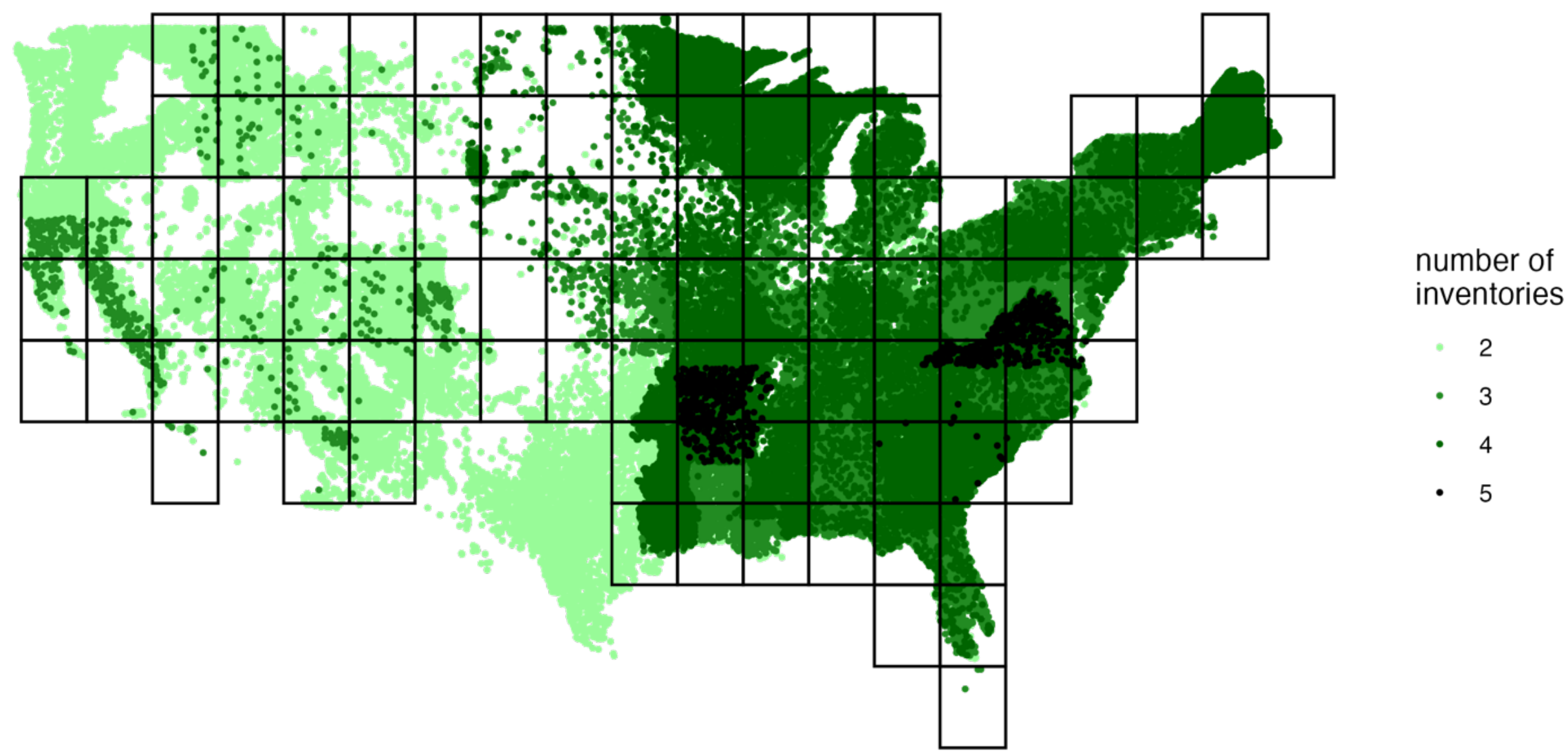

Figure S1: Map of FIA plots used in the study, colored by the number of times each plot was surveyed. All plots shown here were used for most analyses. For the analysis evaluating model performance in space-for-time substitution, only plots that had been inventoried three or more times (primarily located in the eastern US) were used; in one analysis these were grouped into spatial blocks consisting of three-degree grid cells, illustrated here in black.

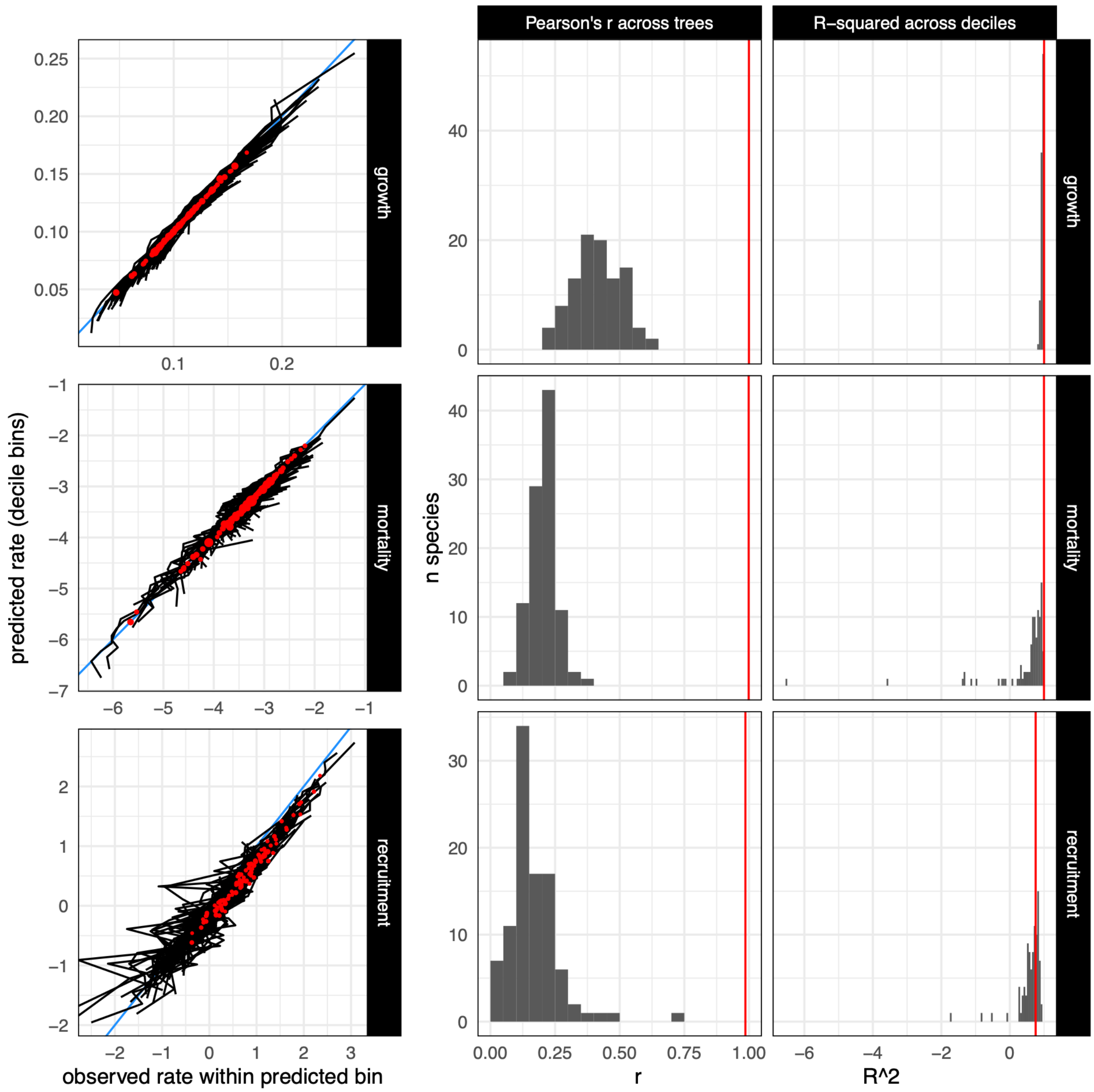

Figure S2: Model fit to training data for each of the three demographic rates. In the scatterplots, every species is represented by a red point indicating its overall mean, and by a black line connecting the ten predictive deciles. The histograms show distributions of species-level correlations between observed and predicted rates across individual trees (*r*, which includes uncertainty form individual-level demographic stochasticity) and coefficients of determination across predictive deciles (R^2, which averages over individual stochasticity to represent population-level demographic rates); red lines indicate *r* and R^2 across species means.

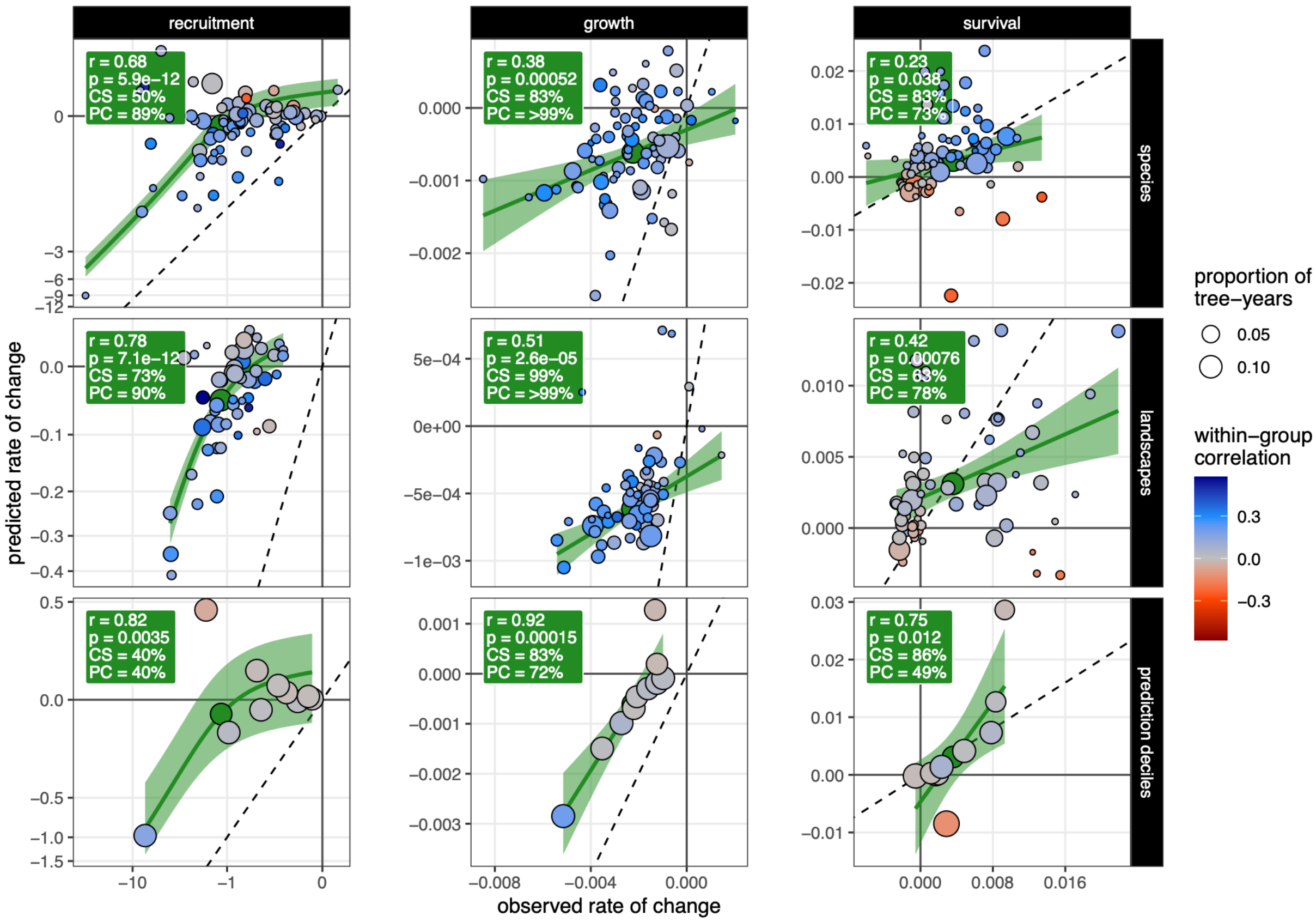

Figure S3: Results of model evaluation for space-for-time substitution, in which observed recent demographic trends (x-axis) were compared to trends predicted by demographic models parameterized on spatial patterns in long-term environmental means (y-axis). Each point represents the mean observed and predicted change for a species (first row), a three-degree spatial block (second row; see Fig. S1 for map), or a decile of the distribution of predicted probabilities (third row), with point size indicating the relative amount of data in each group, and color indicating the correlation between predicted and observed trends across trees within the group. Green lines and shaded regions show linear fits and their 95% confidence intervals across points, while dashed lines are one-to-one references. Annotations are as follows: *r*, correlation across points; *p*, p-value of this correlation; *CS*, percentage of points with the correct sign (i.e. falling in the first or third quadrants); *PC*, percentage of groups with positive correlations across trees *within* the group. Axes for recruitment are shown on a pseudo-log scale (linear near zero, logarithmic beyond) to accommodate the wide range of rates; the green linear fits therefore appear curved.

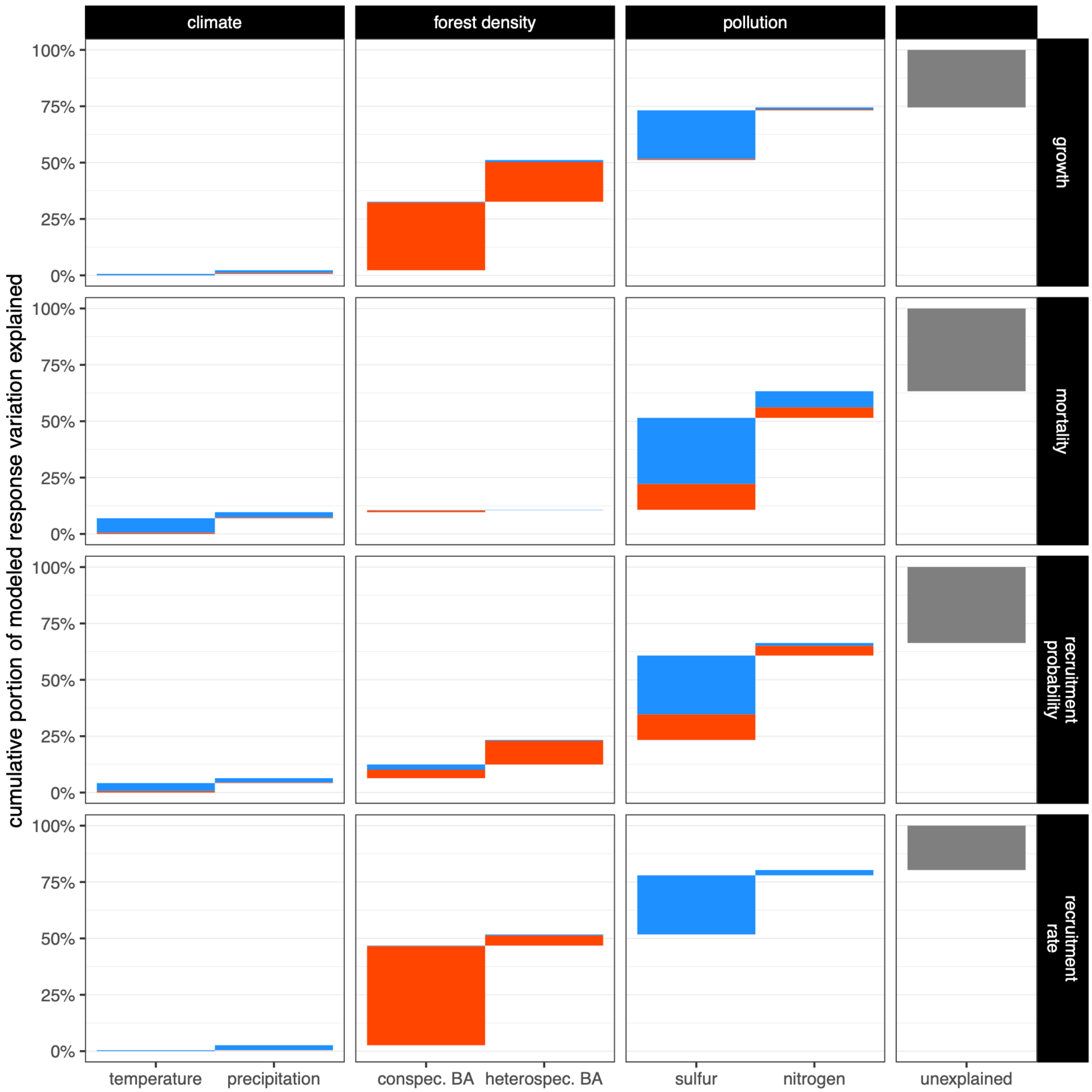

Figure S4: Results of variance partitioning analysis explaining dataset-wide variation in modeled demographic responses (rates of change) as a linear function of variation in sensitivity and exposure. Each of the four rows represents a separate analysis, partitioning the total variation in predicted trends in the demographic rate listed at right into 13 categories (sensitivity and exposure for each of the six environmental variables, as well as unexplained variation).

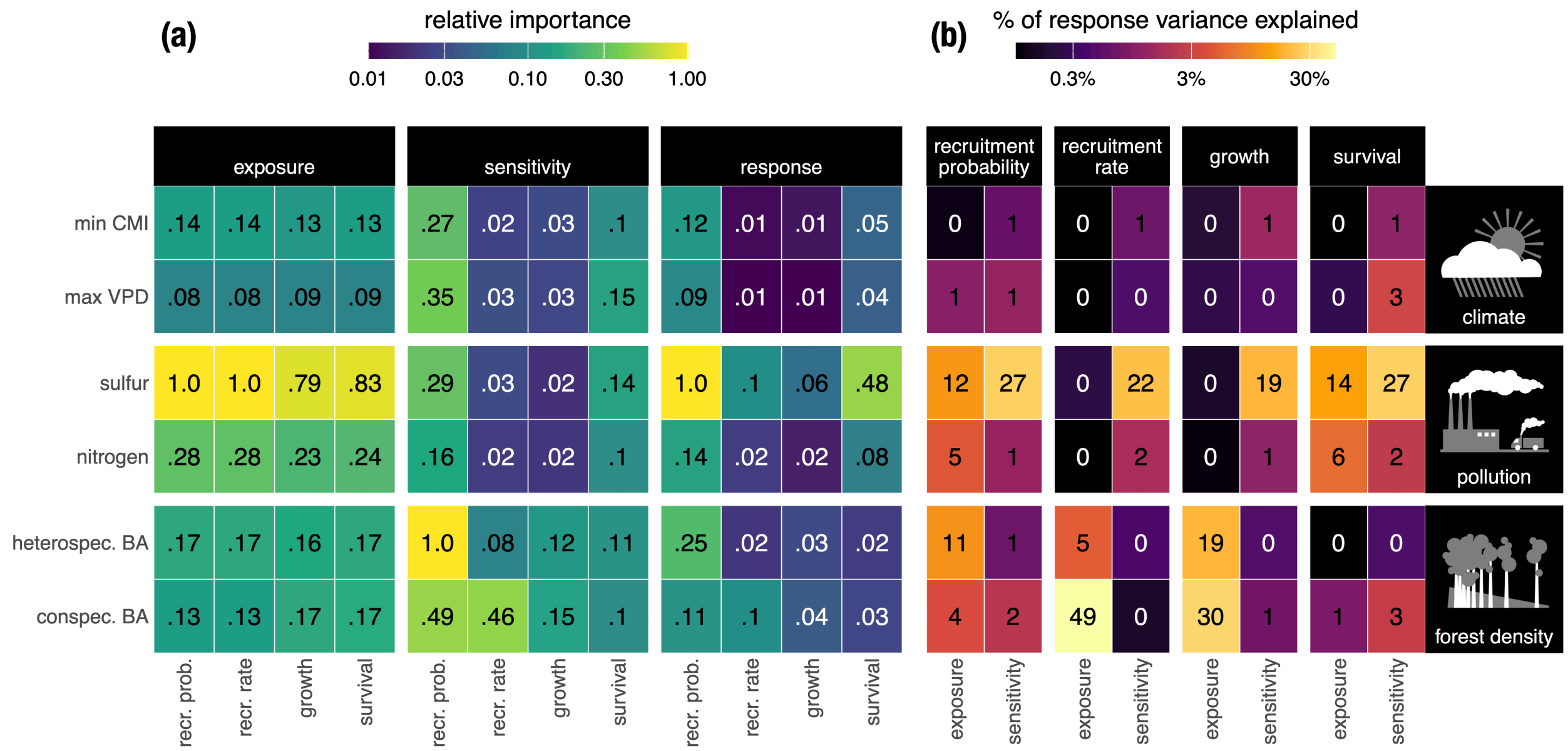

Figure S5: Relative importance of environmental variables in driving modeled demographic trends, from a parallel analysis using an alternative set of climate variables reflecting drought and heat extremes (annual minimum monthly climatic moisture index [CMI] and annual maximum monthly vapor pressure deficit [VPD]) instead of mean annual temperature and precipitation. All other aspects of the analysis are identical to those underlying Fig. 5. (a) Variable importance based on mean absolute effect sizes. (b) Proportion of modeled response variation explained by each variable and component, from variance partitioning analyses; the 12 values depicted under each demographic rate, plus unexplained variance, sum to 100%. Colors are on a log scale. Exposure to maximum VPD is roughly twice that of mean annual temperature, and recruitment probability is more sensitive to these extreme metrics than to mean climate; nonetheless, climate remains the weakest of the three driver categories overall. Note that the two extreme-climate variables are more strongly correlated with each other ($r = -0.68$) than are temperature and precipitation ($r = 0.08$), so their individual contributions in (b) are more diluted by covariation; climate is thus best compared between parameterizations as a category.

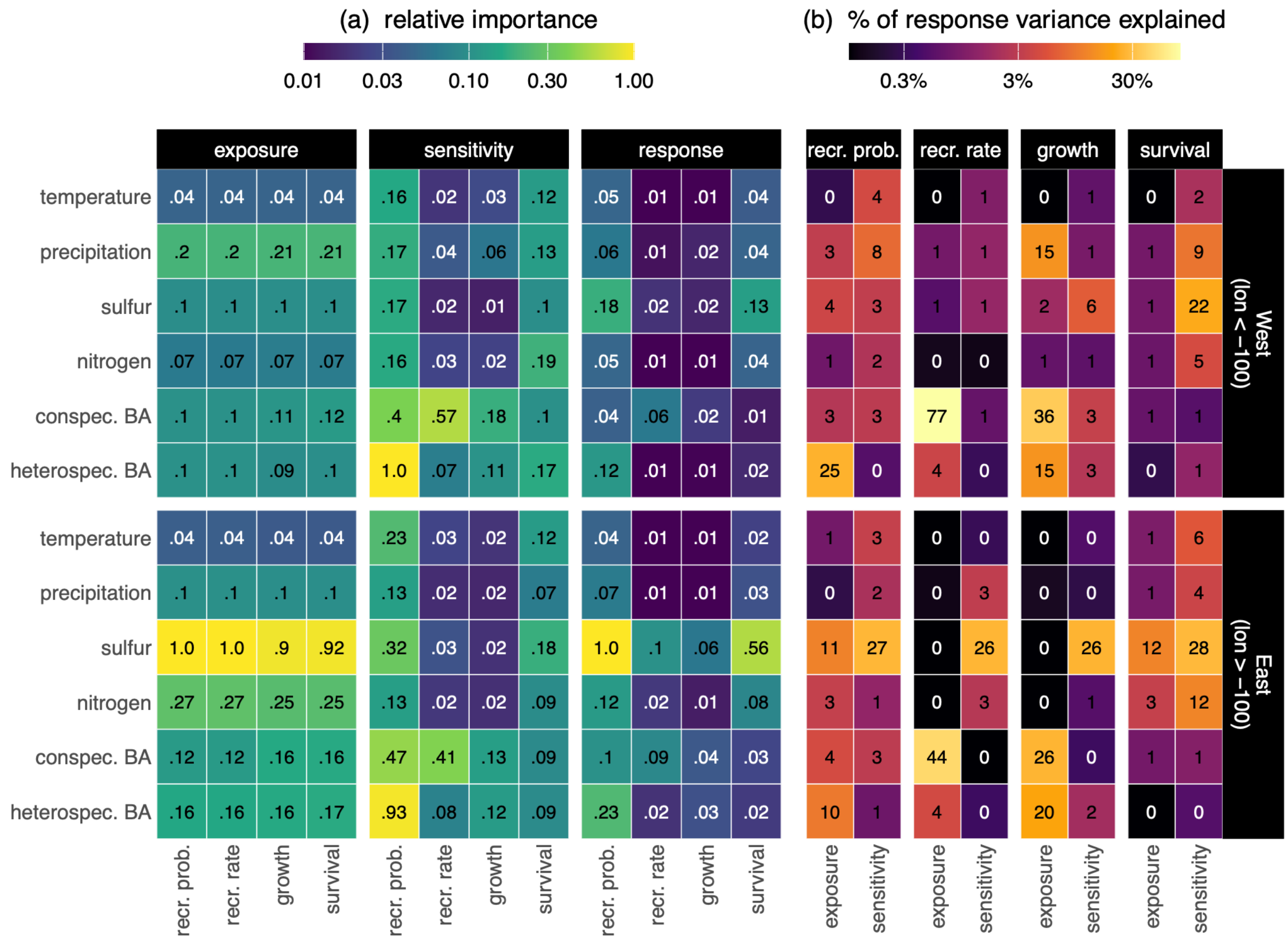

Figure S6: Relative importance of environmental variables in driving modeled demographic trends, computed separately for western and eastern plots. The top row of panels shows results for western plots (west of 100°W) and the bottom row for eastern plots (east of 100°W). (a) Variable importance based on mean absolute effect sizes; values are scaled relative to a single maximum per change component (exposure, sensitivity, response) across both regions, so they are comparable across regions as well as within components. (b) Proportion of modeled response variation explained by variation in each driver and component, from variance partitioning analyses conducted separately within each region; the 12 values depicted under each demographic rate, plus unexplained variance, sum to 100%. Total variance explained differs among regions and demographic rates (ranging from 41% for western survival to 83% for western growth), so values in (b) are most directly compared within rather than across panels; the standardized effect sizes in (a) do not have this limitation. Because the FIA dataset is dominated by eastern plots, results for the East are similar to the combined results presented in Figure 5. Colors are on a log scale.
